# A Limited Number of Amino Acid Permeases Are Crucial for *Cryptococcus neoformans* Survival and Virulence

**DOI:** 10.1155/2024/5566438

**Published:** 2024-08-08

**Authors:** Olufemi S. Folorunso, Olihile M. Sebolai

**Affiliations:** Department of Microbiology and Biochemistry University of the Free State, 205 Nelson Mandela Drive, Park West, Bloemfontein 9301, South Africa

## Abstract

One unique attribute of *Cryptococcus neoformans* is its ability to procure essential monomers from its surroundings to survive in diverse environments. Preferentially, sugars are the energy sources for this opportunistic pathogenic fungus under the carbon catabolite repression (CCR); however, sugar restriction induces alternative use of low molecular weight alcohol, organic acids, and amino acids. The expression of transmembrane amino acid permeases (Aaps) allows *C. neoformans* to utilize different amino acids and their conjugates, notwithstanding under the nitrogen catabolite repression (NCR). Being referred to as global permeases, there is a notion that all cryptococcal Aaps are important to survival and virulence. This functional divergence makes alternative drug targeting against *Cryptococcus* a challenge. We examine the functions and regulations of *C. neoformans* Aap variants with the aim of rationalizing their relevance to cryptococcal cell survival and virulence. Based on nutrient bioavailability, we linked the Cac1 pathway to Ras1 activation for thermotolerance that provides a temperature cushion for Aap activity under physiological conditions. Lastly, mutants of Aaps are examined for significant phenotypic deficiencies/advantages, which buttress the specific importance of limited numbers of Aaps involved in cryptococcal infections.

## 1. Introduction

*Cryptococcus neoformans* remains a highly developed and adaptive opportunistic unicellular fungus capable of surviving in the environment and causing infection in humans/animals. Apart from capsules, melanin, urease, and phospholipase that confer structural and immunodefense features to this clinically important basidiomycete [1–3], it possesses arrays of membrane transporters, permeases, gates, channels, pores, and pumps that allow molecular trafficking of nutrients and metabolites. Even if the nutrients are not readily available to this pathogenic fungus, as observed during systemic infection [4, 5], the robustness of metabolic switch in cryptococcal cells provides evidence of phenotypic adaptation and survival in harsh environments [6–8]. Hence, establishing these forts enables cryptococcal cells to survive starvation, temperature, radiation, oxidation, osmotic stress, salinity, predation, phagocytosis, and pH.

*C. neoformans* also possess multicopy genes encoding redundant and overlapping functional proteins. Among these is the amino acid-polyamine-choline gene family (APC) to which Aap belongs. *Aap* genes encode Aaps that range from Aap1–Aap10 in *C. neoformans* [9]. A further analysis showed that Aap9 and Aap10 are *S. cerevisiae* homologs of Mup1 and Mup3 essential for transporting *S-*containing amino acids with a relative distance to the Aap1–Aap8 protein family [10]. Amino acid permeases are described as secondary carriers and active transporters that allow the uptake of amino acids from the environment via the electrochemical energy gradient mainly supplied by Na^+^/H^+^ ion exchanger and structure–structure interaction between the carrier and substrate [11]. We further speculate that the concentration gradient may also contribute to the driving energy that stimulates membrane nutrient sensors.

Several factors alter the expression of Aaps. Generally, amino acid availability induces specific Aap expression via the activation of yeast Gap2 and SPS-sensing mechanism [9, 12, 13], provided that NH_4_^+^ is unavailable and the CCR is repressed. Environmental nutrient conditions determine the CCR↔NCR switch, which further moderates the Aap expressions. NCR usually activates the expression of the global transcription control factor, Gat1, which further shuts down Aaps but promotes the utilization of environmental nitrogen sources like creatinine, urea, and uric acid for survival and potential virulence [12].

*C. neoformans* express less than half of the total Aaps found in *S. cerevisiae* (24 Aaps) and *Candida albicans* (27 Aaps) to assimilate over 20 types of naturally occurring amino acids and their conjugates [13]. This implies overlapping, synergism, and functional replacement among these limited number of Aaps in *C. neoformans*. Each of the 10 Aaps shows less than 40% sequence homology compared to human Aaps [9]. This suggests an evolutionary advantage to *C. neoformans* in that a small number of Aap variants exist in this fungus, which are redundant and functionally replaceable for survival and virulence. One could think that a smaller number of Aaps in this fungus may enhance functional and regulatory controls with minimal energy input when utilizing alternative nutrient sources. Based on this, drug targets against *C. neoformans* pivotal Aaps will transform antifungal therapy against cryptococcosis, an invasive systemic secondary infection common to immunocompromised and organ-recipient patients.

## 2. Features and Coordination of *C. neoformans* Amino Acid Permeases

Generally, Aaps transport amino acids and their derivatives as a uniporter (rare occurence), symporter (solute-cation), or antiporter (solute-solute) in a proton coupled-dependent mechanism [14]. This proton-gradient force that powers the Aap is mostly generated from the P-type ATPase, such as Na^+^/H^+^ ionic exchanger, which seems to be ATP-independent [15, 16] and *C. neoformans* Aap is encoded by *Nha1* in *C. neoformans*. Like other amino acid transporters (AATs), *C. neoformans* Aap contains 9–12 putative membrane-spanning domains. The H_2_N– and –COOH polypeptide end termini of Aaps are embedded in the cytoplasmic phase, an attribute that appears contrary to most transmembrane protein topology [17, 18]. *C. neoformans* Aaps display various features like the yeast AAT family, including overlapping, homology, redundancy, replacement, synergism, and polycistronic expression [18–20].

Largely, Aap1, Aap2, Aap3, Aap4, and Aap5 appeared clustered with higher percentage of sequence homology than Aap6 and Aap7, which shared 41.1–41.4% [9, 21]. Functionally, Aap1 and Aap2 share 80.9% similarity in amino acid uptake, and compared to Aap3, the three Aaps share 49% [9, 21]. Aap4 and Aap5 share about 89.5% similarity and overlap in the types of amino acids transported [9]. Aap8 is the most diverged permease among others [21]. High (Mup1) and low (Mup3) affinity Met permeases (corresponding to Aap9 and Aap10) share 16.5% amino acid similarity and are significantly involved in *S-*amino acid uptake (Table 1). In fact, because of these features, each of these pairs is referred to as global permease. Fernandes et al. referred to Aap1–Aap8 as global permeases as well [21]. In our view, the use of global permease appears permissive, and we realized that a permease becomes global once each class of amino acid shows a repressive/inductive growth in the absence/presence of such permease. Regardless of the condition, Aap2–Aap8 are the most expressed permeases in nondextrose synthetic media compared to the rich media [21].

Regarding selectivity, little is known about Aap affinity for specific amino acids in *C. neoformans*. However, earlier work had shown that plant-based Aap genes expressed in yeast showed selective affinity [22]. Aap1, Aap2, Aap3, Aap4, and Aap5 showed low selective affinity towards amino acid side chains. Aap3 and Aap5 effectively transport basic amino acids but generally display AAT attributes *in vivo*, and Aap6 had the highest substrate uptake compared to other Aaps [23]. A few reports on the *C. neoformans* Aap specificity had shown that Aap3 appeared more specific to Lys than Aap2 [24] (Table 1), which explains a complementary effect of *Δaap5* mutant cultured in Lys media [19]. Among the Aaps, Aap2 shows the highest broad specificity towards tripeptides in culture media, but Aap4 and Aap5 are mostly expressed in this medium (Table 1). Surprisingly, Aap4 but not Aap5 is solely responsible for acidic amino acid uptake in supplemented alkaline synthetic dextrose (SD) media at 30°C. This explains why *Δaap4* growth was significantly affected in *L*-Asp, *D*-Asp, and *L*-Glu [19].

**Figure 1:**
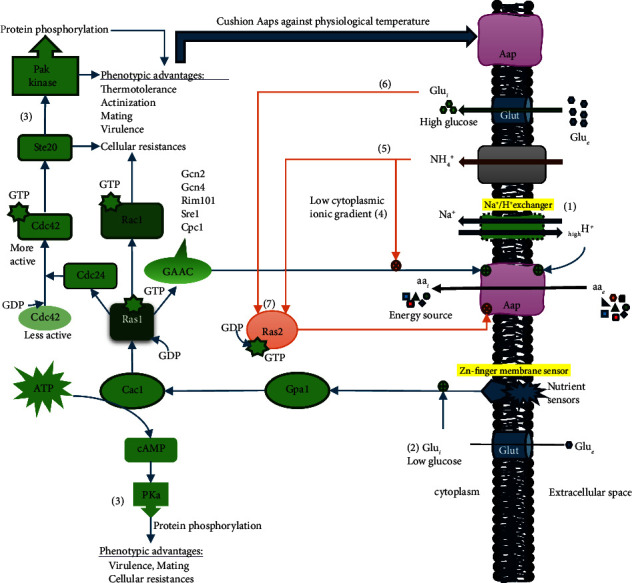
Schematic illustration showing Aap directly driven by the Na^+^/H^+^ exchanger and further supported by the solute concentration and ionic gradients, low intracellular glucose, and Ras1 cascade events. Enhanced activity: (1) Na^+^/H^+^ ionic exchanger provides a proton gradient and ΔpH that drive Aap, (2) low intracellular glucose (Glu_*i*_) activates Gpa1, orchestrating the Cac1 activity to convert ATP to cAMP when energy is needed. The desperate need for energy may favor the activation of Ras1 protein via the GTPase activity that converts GDP to GTP. Activated Ras1p is a multifunctional factor that activates other regulatory factors. One of them is GAAC factors that sense environmental stress and initiate amino acid biosynthesis and uptake via a proximal promoter element of polycistronic genes [18], (3) formation of Pka, Ste20, and Pak1 via Ras1 activation promotes a series of phenotypic advantages, such as thermotolerance and actinization (enhanced cytoskeleton), which supports Aap stability for continuous amino acid uptake, even at nonpermissive temperature. Reduced activity: (4) low concentration/ionic gradient will limit the Aap activity, (5) presence of NH_4_^+^ (NCR) will repress Aap activity via Gcn-translating ribosome complex, (6) high level of Glu_*i*_ via the glucose transporters, like Glut1, initiates CCR that shutdown Aap activity via a conserved repressor consensus sequence typical to the *Aap* promoter site [18], (7) high level of intracellular NH_4_^+^ or Glu_*i*_ enhances the activation of Ras2p in the presence of GTP. Activated Ras2p does the opposite of Ras1 regarding Aap activity. Pak–p21-activated protein kinase, which includes Ste20*α*, Ste20a, and Pak1; Cdc24—a GEF protein mediating a conserved signalling event for thermotolerance, polarized growth, and pathogenesis via Ras1, Cdc24, Cdc42, and Pak kinase Ste20; GEF–Guanine nucleotide exchange factor facilitating the GTPase activity in GDP↔GTP conversion; Rac1 and Cdc42–effectors for vacuole/endosomal morphology and actin organization; Ras1–GTPase involved in G-protein signalling in the adenylate cyclase-activating pathway; Cac1–*Cryptococcus* adenylyl cyclase; Pka–cAMP-dependent protein kinase A catalytic subunit; and Gpa–G-protein *α* subunit.

Intracellular glucose and NH_4_^+^ levels play a significant role in regulating the expression of *Aap* genes; hence, Aap activity is constantly under the control of CCR and NCR (Figure 1). Though the NCR selectively represses Aap, CCR appears to repress all the Aap expression in the presence of glucose (preferred carbon source) compared to galactose (alternative carbon source) (Table 1). The transcription analysis further showed that NCR could repress Aap2, Aap5, and Aap8 in the presence of NH_4_^+^ but not Aap4, which means Aap4 is promiscuous to most nitrogen sources, including *D*-Asp; however, Aap3 and Aap7 remained minimally expressed [21] (Table 1).

The involvement of SPS-sensing (Stp1 and Stp2) homologues in *C. neoformans,* Znf2 and Zap1, respectively, failed to produce any relevant significant growth defect with respect to *Δznf2* and *Δzap1* mutant growth studies in amino acid-supplemented SD media when compared to the H99 *wt* [9]. As shown in Figure 1, alternative membrane zinc finger sensors may exist in *C. neoformans* to detect amino acids in the surrounding media when the intracellular pool is depleted, reminiscent of the complementary pathway to the limited number of Aaps.

In exploring how Aaps are coordinated, Ras signalling was identified as one of the pivotal controlling cascades that regulate Aaps activities [9] (Figure 1). Our previous publication suggested that a positive interaction between Cac1 and Ras1 may exist [25]. Though the Gpr4 protein that senses Met from the environment may be absent in *C. neoformans,* unlike the yeast; however, some yet unknown zinc finger homologue sensors may be present in cryptococcal cells, which are induced for amino acid homeostasis. So, if this interaction exits, then the metabolic and energy balance functions of cAMP/Pka orchestrated by Cac1 may be perceived by Ras1 protein, which will activate GAAC transcription factors to sense the intracellular amino acid levels and accordingly induce the expression of polycistronic *Aaps* in the absence of NH_4_^+^. Increasing levels of intracellular glucose and NH_4_^+^ enhance Ras2p-GTPase activity. Activated Ras2p reduces Aap activity contrary to Ras1p (Figure 1).

## 3. Nitrogen Sources Induce GATA Transcription Factors via Nitrogen Catabolite Repression (NCR) and Determine the Expression of Amino Acid Permeases (Aaps)/Transporters (Amt) in *C. neoformans*

Preferentially, NH_4_^+^ compounds are the sole nitrogen source for cryptococcal cells via uptake by a pair of transmembrane proteins called ammonium permease/transporter, constitutive Amt1 (low affinity) and inductive Amt2 (high affinity) [26]. The presence of NH_4_^+^ induces NCR, whereby every other nitrogen source is unexplored by the cell. With NCR, certain nitrogen sources are poorly assimilated, especially the charged aliphatic amino acids, such as Leu, Ile, and Val, perhaps because of the energy cost metabolism of their side chain hydrocarbons. Others like Ala, Cys, Thr, and His hardly support *C. neoformans* growth (Table 2). More importantly, NCR activates the GATA transcription factor that further reduces Aaps activation but promotes NH_4_^+^ permeases.

Fungal GATA-transcriptional factors are specific groups of transcriptional regulatory factors (such as Gat1, Gat5, Gat6, Gat201, Gat204, Bwc2, Bzp2, Cir1, Rim101, Sit1, and Tup1), which are usually characterized by tetra-Cys (Cys-X-X-Cys-X_17-20_-Cys-X-X-Cys-5-12) basic residues DNA-binding domain recurring zinc finger motif that allows specific promoter site interaction for activating transcription factors necessary for “on/off” expression of genes that facilitate virulence, adaptation, survival, mating, and nutrient uptake [12].

Generally, GATA transcription factors recognize T/A-GATA-Α/G DNA sequence on the *Aap* promoter site via their DNA-binding domain [18]. This interaction, which could involve more than one GATA factor, either induces/represses Aap expression as dictated by NCR (Table 3). Unfortunately, not much work has been done on cryptococcal GATA-Aap interactions; the present data shows that the focus has been on Amt1, Amt2, Aap4, and Aap5 (Table 3). Each GATA member plays a significant role in regulating nutrient uptake, virulence, mating, fruiting, morphology, and resistance, but more work is needed to unravel how these are linked to specific Aap. Morphologically, Gat1 expression favors the reversible pseudohyphal formation mostly observed with *C. neoformans* serotypes A and D in a low nitrogen environment as found within the amoeba intracellular region. This condition induces the expression of Amt1 and Amt2 for the uptake of limiting NH_4_^+^ [34]. Though there is evidence of conditional pseudohyphal ⟷ yeast formation under nitrogen-limiting conditions, only the yeast state is convincingly virulent and disseminating [35–37]. This shows that the conditional nitrogen level determines the cryptococcal cell morphotypes, which could contribute to survival and virulence. Recognizing this morphological transition under a limiting nitrogen source, Amt2 is therefore proposed as a fungal NH_4_^+^ sensor that induces signalling events towards survival and mating in response to nutritional levels [26].

Gat1 is usually repressed to promote NCR during thermotolerance—a functional control phenotypic virulence of *Cryptococcus* under the stress-induced Ca^2+^-orchestrated Cna1-Crz1 pathway [25]. Surprisingly, Gat1 enhances the activity of Cdk-related kinase (Crk1) that inhibits Mat2 from controlling filamentation and bisexual mating in cryptococcal cells [38, 39]; however, Cna1 activity is low under this condition. As stated earlier, nonpermissive temperature represses Gat1 expression. This repression limits Crk1 activity but elevates Cna1 activity to promote a series of functional protein dephosphorylation that promotes cell wall integrity for capsule/melanin formation, thermotolerance, and even sexual reproduction via Crz1, Lhp1, Puf4, and Pbp1 dephosphorylation [40]. Thus, Gat1 enables *Cryptococcus* to determine the shift between phosphorylation-dependent and dephosphorylation-dependent activated proteins for survival, mating, and virulence.

Nutritionally, keeping NCR under repression by Gat1 is necessary to facilitate the utilization of other nitrogen sources like urea, urate, and creatinine. In doing so, it negates basidiospores, melanin formation, and thermotolerance but favoring capsule formation. This was well demonstrated by Lee et al. [12] that capsule formation increased considerably in strain H99 when cultivated in a medium containing Asn, urea, urate, or creatinine but not Gln, Pro, or Ala-containing medium. This capsule formation hitherto supported in amino acid-supplemented media is prosaically affected by introducing NH_4_^+^ into the media, which now favored melanin formation [12]. So, *in vivo* virulence of cryptococcal cells must entail repressed Gat1. Lee et al. further showed that *Δgat1* mutant displayed slightly more virulence compared to the wild-type *(wt)* in a murine inhalation/infectious model of cryptococcosis (MIMC). This indicates that NCR differentially regulates virulence factors under relieved Gat1 and that certain amino acids can override the NCR effect in the dire need for virulence.

Relative to NH_4_^+^ as the preferred nitrogen source at 30°C, Val, Ile, and Met-containing synthetic dextrose (SD) media poorly support the growth of *C. neoformans,* but Leu, Ser, Lys, and Phe are better nitrogen sources to activate Aaps (Table 2). Furthermore, Gly, Asp, Asn, Glu, Gln, Arg, Trp, and Pro highly compete with NH_4_^+^ in culturable amino acid media. At 37°C, Val and Met poorly support *C. neoformans* growth in SD media, but Gly, Leu, Ile, Ser, Trp, and Phe are good nitrogen sources, while Asp, Asn, Glu, Gln, Arg, Lys, and Pro are better nitrogen sources (Table 2). *L*-Tyr failed to dissolve at permissive pH for culturing *C. neoformans*; hence, it is unsuitable to be tested as a nitrogen source in *C. neoformans* experimentation. Therefore, basic and acidic amino acids are the top players that activate Aaps. This perhaps correlates with the pH-dependent antiporter/symporter Aaps.

Speculation of decreased Aap conformation with increased temperature (30 ⟶ 37°C) may be responsible for inefficient amino acid uptake, as most growths in all amino acids, except Asp, appear significantly less than NH_4_^+^ in SD at 37°C [13]. However, as shown in Figure 1, the expression of Ras1p and its dependent accessories, like Rac1, Ste20, Pak1, and GAAC proteins, may modulate temperature-sensitive Aaps in thermotolerance vis-à-vis the global thermotolerance role of Cna1-Crz1 activation, which occurs as nonpermissive temperature represses Gat1.

## 4. Specific Deletion of *Aap* and Their Regulatory Genes Attenuates *C. neoformans* Survival and Virulence

Generally, *Δaap1Δaap2* mutants are thermosensitive (at 37°C in amino acid-supplemented SD media), hypocapsulated, and hypovirulent in the *Galleria mellonella* infection model [9]. Mutants such as *Δaap1*, *Δaap2*, *Δaap6*, *Δaap8*, and *Δaap1Δaap2* failed to display any significant difference in growth when compared to the *wt* at 30 or 37°C in SD supplemented with amino acids/NH_4_^+^ or in YPD. This suggests that cell growth and morphology are not affected by the absence of any of these Aaps in solid media. In liquid culture, however, growth reduction was observed in *Δaap1* in the presence of Met or Pro at 30 or 37°C [9]. Also, a liquid culture of *Δaap1Δaap2* mutant containing Gln or Arg displayed growth reduction at 30°C, and *Δaap8* mutant showed growth reduction in media containing Met, Glu, and Trp [9]. By deduction, if *Δaap1* and *Δaap8* displayed reduced growth in Met, one could have expected either of the mutants to grow well in Met medium if synergism or transporter replacement exists between Aap1 and Aap8. So, this shows that Aap far from each other on allele may not replace or support each other in nutrient uptake, an attribute related to relative specificity and affinity for substrates.

Up to 60% of all culturable amino acids in supplemented SD appeared not to support growth in *Δaap1Δaap2* and *Δaap4Δaap5* mutants at 37°C. In terms of virulence, Aap1, Aap2, Aap6, and Aap8 appeared nonessential because mutants of these genes, including *Δaap1Δaap2*, failed to show any significant difference in the mating, filamentation, melanin expression, PLB, and urease activities when compared to the *wt* [9]. Apart from being nonessential for virulence, none of the mutants seemed affected by oxidative stress agents, alkaline conditions, osmotic/salt stress agents, and cell wall stress agents at 30°C or 37°C. Surprisingly, unlike the single mutants (*Δaap1*, *Δaap2*, *Δaap4*, *Δaap5*, *Δaap9*, and *Δaap10*), the double mutants *Δaap1Δaap2*, *Δaap4Δaap5*, and *Δaap9Δaap10* displayed reduced capsule size at 37°C when compared to the *wt*. However, the capsules are similar at 30°C, and the virulence of only *Δaap1Δaap2* and *Δaap8* mutants was attenuated in *G. mellonella* [9].

From Martho et al. *Δaap2*, *Δaap4*, *Δaap5*, *Δaap9, Δaap10*, and *Δaap9Δaap10* showed no sign of attenuated virulence in *G. mellonella,* but *Δaap4Δaap5* mutants are hypovirulent at 30 or 37°C [13]. Furthermore, virulence investigation in MIMC showed that mice inoculated with the *wt*, *Δaap4*, and *Δaap5* died within a month, while the mice inoculated with *Δaap4Δaap5* mutant survived with viable recovery colonies from the lungs and livers [13]. In addition, *Δaap2*, *Δaap4*, *Δaap5*, and *Δaap4Δaap5* mutants displayed *wt* phenotypic sensitivity to AmpB, hypersensitive to FCZ, and hyper-resistant to eugenol due to the absence of specific Aaps. Deletion of *Aap4* slightly affects cryptococcal cell growth in Asp, Glu, and Phe at pH 7–9 and 30°C, whereas deleting *Aap5* slightly affects the growth in Phe and Lys only [19]. This shows that Aap4 and Aap5 are important permeases for thermotolerance, antifungal resistance, oxidative stress response for tissue invasion, survival, virulence in the animal model, and alkaline resistance. By supporting other published works, these Aaps are potential targets for antifungal therapy [41]; notwithstanding, the compensatory effects of the Aaps cannot be ruled out, especially those with close homology.

Gat1, Gat5, and Gat6 are the most studied GATA-transcriptional factors. Deletion of *Gat1*, among other GATA transcription factors, promotes poor to no growth of *C. neoformans* in most nitrogen sources except Pro and, to some extent, Arg because these two amino acids are readily converted to Glu [12]. Single deletion of other GATA family genes (*Δgat201*, *Δgat204*, *Δbwc2*, *Δbzp2*, or *Δcir1*) failed to shut down fungal growth in all the commonly used nitrogen sources [12]. This means various Aaps may still be activated for amino acid uptake under a single GATA gene deletion except in *Δgat1* mutant. However, in addition to *Gat1/Are1* deletion, specific deletion of *Gat5* and *Gat6* drastically endanger cryptococcal cells to stress, radiation, and antifungal attack because *Amt1*, *Amt2*, *Gdh1*, *Aap4*, and *Aap5*, identified as downstream controlled genes to GATA, are differentially affected [22, 27].

The growth defect of *Δgat1* strain H99 mutant in urea, urate, or creatinine-containing media with reduced expression of genes involved in nitrogen metabolism (such as *Gdh1*, *Amt1*, and *Amt2*) is a serendipitous observation that contradicts the ecological niche of *C. neoformans.* Ironically, a similar survival growth observed with the *wt* and *Δgat1* mutants cultivated in pigeon guano media is an indication that the surviving *Δgat1* mutant in the same environment with the *wt* poses an investigation into the unique nitrogen sources suitable for such a mutant. One better explanation may be that the pigeon guano media are rich in Pro and Arg, as inferred from Lee et al. observations [12]. Not only this, others have also observed the growth of *Δgat1* mutant in Pro, Arg, urea, and NH_4_^+^-containing media [42, 43]. This shows that Pro and Arg might just be sufficient to produce a minimal required energy source for *C. neoformans* survival.

Gpp2 (glycerol-3-phosphate phosphatase) is another Aap controlling factor whose deletion induces a significant number of Aap genes especially *Aap4* and *Aap5*, but downregulates sulfur-containing amino acid biosynthesis (Met and Cys) [44]. Similarly, high-osmotic glycerol mutant (*Δhog1*) had shown elevated expression of *Aap4* and *Aap5* [45]. Consequently, *Δgpp2* and *Δhog1* mutants are susceptible to eugenol, which has hitherto shown to be resistant in *Δaap4* and *Δaap5* mutants [44, 45].

Ras1 protein is another regulatory factor of most *Aap* transcriptions. Ras1 expression produces a series of Ras proteins that bind GTPases for GTP-activated Ras proteins, which are involved in the Ras1/MAPK pathways during thermotolerance [25]. Since Aaps are nearly defective at nonpermissive temperatures, expression of Ras1p appears as a surviving strategy by *C. neoformans* at physiological temperature (Figure 1). Consequently, this enhances the functional stability of Aaps towards the uptake of growth-supporting amino acids. It is yet unrevealed how *C. neoformans* is able to couple Ras1p to Aap expressions; however, we speculate that this interaction may not be contingent on transcription factor-induced expression but rather an indirect outcome of Ste20 and Pak1 kinase activities via Ras1 ⟶ Cdc24 ⟶ Cdc42 pathway (Figure 1), which are involved explicitly in actin/cytoskeletal regulation, cell wall, and membrane integrity [25]. It is reasonable to infer that if these three phenotypic advantages are in place, then membrane transporters, like Aaps, will function efficiently even under nonpermissive temperatures and membrane stressors.

Calvete et al. demonstrated that *Δras1* mutants showed poor growth in about 73% of all culturable amino acid-supplemented SD media at 30°C but a *wt* growth in Ser, Lys, Trp, and Ile, which are mostly “good” amino acids promoting fungal growth (Table 2). Interestingly, the growth-supporting amino acids reduced to 46% in this mutant at 37°C, with significant growth reduction in all the tested amino acids compared to the *wt* [9]. At these temperatures, there was a significant reduction in the expression of *Aap1*, *Aap2*, *Aap4*, *Aap6*, *Aap8*, *Aap9*, and *Aap10*, but only *Aap5* transcript matched the *wt* expression at 37°C in SD media supplemented with Trp, His, and Met [9].

The indirect effect of the Ras1 signalling on Aap function reiterates the role of Ras1/Ste20 signalling pathway in the nutritional balance of *C. neoformans* against thermal stress. Thermotolerance is critical to survive alternative nutrient sources, maintain cellular morphology, and exhibit virulence. Just as Aap4 and Aap5 were identified as potential drug targets against cryptococcal infection, the unique region of sequence homology within the regulatory domain of Ras1 protein across a few numbers of pathogenic yeast has also been speculated as a drug target [46].

## 5. *C. neoformans* Differentially Expressed *Aap* Based on Conditional Nutrient

Among the ten variant Aaps expressed by *C. neoformans,* as shown in Table 1, *Aap2*, *Aap4*, *Aap5*, *Aap6*, *Aap8*, *Aap9*, and *Aap10* are more differentially expressed in the amino acid-supplemented SD minimal media than the rich media (YPD) [21, 47]. Without NH_4_^+^ (a preferred nitrogen source), global/general amino acid control (GAAC) is triggered by nitrogen starvation, leading to the preponderant production of amino acid biosynthetic enzymes in the yeast. For example, nitrogen starvation induces Trp biosynthetic enzymes encoded by *Trp2*, *Trp3*, *Trp4*, and *Trp5* [21], but how *C. neoformans* are able to switch between the need for biosynthesis and Aap induction in amino acid limiting conditions remains unclear. The ATP-independent powering of Aaps via Na^+^/H^+^ exchanger appears to be metabolically and economically feasible for the fungi rather than ATP-dependent biosynthesis. Nevertheless, at the need for critically essential amino acids, salvage pathways could be preferred at the expense of assimilated amino acids rather than *de novo* biosynthesis. Thus, depending on the nutrient bioavailability, *C. neoformans* may switch between the need for amino acid biosynthesis and uptake.

By looking at the phenotypic characteristics of *Aap* mutants, it appears that *Aap1*, *Aap2*, *Aap6*, and *Aap8* may not be very critical to the survival of *C. neoformans* because mutant of each gene shows no significant difference when compared to the *wt* cultured in supplemented SD or rich solid media at 30 or 37°C [9]. Further investigation revealed that *Δaap1* may be sensitive to Met and Pro supplemented SD at 30°C in the same way *Δaap1Δaap2* mutant was sensitive to Gln and Arg [9]. In addition to *Δaap1Δaap2*, the proportion of amino acids that failed to support significant growth of *Δaap9Δaap10* mutant appeared to increase from 30 to 37°C; however, this is less significant when compared to the immense number of amino acids that failed to support *Δaap4* and *Δaap5* growth under the same conditions (Table 4) [9, 13]. Therefore, *Aap4* and *Aap5* genes appear highly critical to the survival of *C. neoformans*.

“Aaps” and “Amts” are a family of APC-superfamily membrane transporters/permeases involved in the adhesion and aggregation of *C. neoformans* cells. Infrequent in humans and mice, low nitrogen in amoeba promotes the pseudohyphal morphotype of *C. neoformans*, which is highly contributed to by Amt1 and Amt2 permeases with improved agar adherence and invasion [34, 48] (Table 4). This phenotypic advantage may contribute to infection latency usually observed in infected humans. Nevertheless, infection latency is a result of yeast ⟷ titanisation morphotype caused by various factors like nutrient deprivation, host immune attack, hypoxia, pH, CO_2_, and antifungal exposure as controlled by multiple regulatory pathways, such as cAMP/Pka, Qsp1/Qsp2 (quorum sensing), Rim101, Pkc1, Sod1/Sod2 redox response [49–52]. It is important to note that the significant contribution of GATA and Aaps to *C. neoformans* titanisation is a unique yet unexplored area. We hypothesize that Bzp2, Gat5, Gat6, Gat201, and Rim101, which are mostly involved in the processes that cautiously promote titanisation, contribute to this cell morphotype. This may invariably promote Aap4 and Aap5 as downstream regulatory permeases to titanisation.

Further into the surviving strategy, a consortium of cryptococcal cells could aggregate to form cryptococcomas, a biofilm-like aggregate of cryptococcal cells crucial for tissue survival, invasion, and colonization [53]. In this state, metabolic processes, replication, translation, and membrane transporters/permeases are generally repressed to minimize energy-demanding cellular activities [54]. This means that most Aap activities are repressed in biofilm formation. It is important to stress that cryptococcomas is a nutrient-depended surviving morphotype involving capsular component formation, adhesion molecules, urease, and laccase activities, which are jointly coordinated by GATA and other transcription factors [30, 55–57]. In this case, the expressions and activities of Aap4 and Aap5 may be suspected because the expressions of the two Aaps are coordinated by GATA genes [22, 27].

The limiting glucose level in macrophage phagolysosomes is an initiating condition for Aaps and Amts expressions, much more the intracellular amino acid deficient in neutrophils and macrophages. Similar to engulfed *S. cerevisiae* and *C. albicans* [58, 59]*, C. neoformans* may be provoked for Aap expression towards amino acid uptake and alternative carbon metabolisms when phagocytosed by immune cells [60]. As stated earlier, Amt1 and Amt2 are involved in pseudohyphal formation for chances to survive amoeboid killing [36] and perhaps, phagocytosis; however, *Δamt1Δamt2* mutants have been reported virulent, let alone individual mutant [26]. This shows that the condition for surviving phagocytosis is not contingent on pseudohyphal formation. So, surviving macrophage attack entails concerted effects of different Aaps, especially Aap4 and Aap5 (which are expressed for acidic amino acids uptake), in addition to other key morphogenesis transcription factors like Znf2 and RAM proteins (such as Tao3, Kic1, Cbk1, Mob2, and Sog2) [35].

Regardless of the temperature, lack of one variant of Aap or distantly related variants may not affect the cryptococcal virulence; however, double mutation of closely related variants may affect capsule formation at nonpermissive temperatures. The most significant attenuated virulence and antifungal sensitivity reported for *Aap* mutants are found in *Δaap4Δaap5* mutant [9]. This shows the critical roles of Aap4 and Aap5 in most of the phenotypical advantages that contribute to the survival, resistance, and infection caused by *C. neoformans*.

## 6. Aap4 and Aap5 Are Essential for the Survival and Virulence of *C. neoformans* among Other Aaps

The functions of Aap4 and Aap5 in the survival and virulence of *C. neoformans* are remarkable. These two integrated membrane transporters share the highest percentage of sequence homology among other Aaps with significant overlapping functions. This may be responsible for their redundant functions concerning amino acid affinity and uptake. From Table 4, it could be deduced that the lack of one gene may be complemented by the other, but the absence of these two *Aap* genes is deleterious and significantly impacting cell growth, morphology, survival, mating, fruiting, and virulence.

In addition to being a global permease, Aap4 and Aap5 promote thermotolerance and response to oxidative stress, and the growth of the double mutant is significantly impacted from 30 to 37°C in a single amino acid medium or the presence of ≥5 mM H_2_O_2_ (Table 4). Neither Δaap4 nor Δaap5 mutant is affected by pH or salt solution; however, *Δaap4Δaap5* mutants showed a substantial growth defect at 37°C, which appeared to be restored as pH increased gradually into the alkaline state or when supplemented with 0.75 M NaCl (a condition that generates H^+^ via Na^+^/H^+^ antiporter that drives other amino acid permeases to compensate for the deletion of *Aap4* and *Aap5*) albeit little restoration compared to the *wt* [9, 13, 61].

Compared to other Aaps, Aap2, Aap3, Aap9, and Aap10 are regarded as minor permeases, while Aap4 and Aap5 as major permeases. Besides, Aap1, Aap2, and Aap3 have also been tagged “global permeases” [9, 13, 61]—description, which seems to be arbitrarily assigned to all *C. neoformans* Aaps, including Aap9 and Aap10 [9, 21]. Aap9 and Aap10 appear highly competitive with Aap4 and Aap5 in their function as global permeases with functionally high and low substrate affinity, respectively. Deletion of *Aap9* and *Aap10* drastically impaired cryptococcal thermotolerance (Table 1).

From a general perspective, stress and virulence assays showed that all *C. neoformansAap* single mutants are not remarkably subjected to peroxide stress or affected by high temperatures in rich media, irrespective of the amino acid components of the synthetic dextrose (SD). However, a double mutant strain *Δaap4Δaap5* is thermosensitive and will not withstand oxidative stress. This doubly mutated strain also shows weak growth in amino acid culture media (Table 1). The significant growth defect of *Δaap4Δaap5* mutants at 37°C in YPD or SD shows that the two permeases (or at least Aap4) are essential for thermotolerance [9, 13, 61].

In addition, extensive sequence analysis has shown that *Aap4* and *Aap5* are the most significantly and differentially expressed downstream genes connected to GATA—Gat5 and Gat6 in the same way Gat1 controls *Amt1* and *Amt2* expressions. Though Gat5 and Gat6 may not directly contribute to virulence, their effect in upregulating *Aap4* and *Aap5* transcriptions under depleted nutrients is evidence of cellular desperation to survive harsh environments [27]. Not only this, *Δgat5* and *Δgat6C. neoformans* mutants exposed to irradiation under nitrogen depletion showed a significant expression of *Aap4* and *Aap5*, which indicates evidence of nitrogen scavenging activity of the mutants to survive and repair radiation damage [27].

The virulence of *C. neoformans* depends on various factors. Beginning with capsular size, all single gene deleted *Aap* mutants appeared not to be affected in capsule formation; hence, they are virulent in *G. mellonella* and MIMC. However, a limited number of double gene deleted mutants displayed varying virulence. For example, *Δaap1Δaap2* mutants are avirulent in *G. mellonella,* while *Δaap8* mutants are hypovirulent. Interestingly, *Δaap4Δaap5* mutants are the only avirulent mutants in *G. mellonella* as well as in MIMC [13].

There are a few but diverse works on the function of Aaps in *C. neoformans.* The endless conditional variability at which each Aap is studied made it difficult to balance the *C. neoformans* survival, virulence, and Aaps. Table 2 shows different conditional variability at which *C. neoformans* Aaps are studied. Careful consideration of this table indicates that Aap4 expression appears not under the NH_4_^+^ superior regulatory control and because Aap4 shares 89.5% sequence homology with Aap5, thus suggesting a remarkable combined contribution of these two redundant but critically important permeases to the survival and virulence of cryptococcal cells.

There are quite a few competitive attributes between *Δaap1Δaap2* and *Δaap4Δaap5* mutants. The dual mutated strains are thermosensitive, characterized by poor growth in most culturable amino acid media. Each could compensate for the other; hence, the observed defects in the single mutated strain are less compared to the double mutated strain. These attributes are also confirmed with *Δaap9Δaap10* strain [9]. Further consideration showed that *Δaap1*, *Δaap2*, *Δaap1Δaap2*, *Δaap4*, *Δaap5*, *Δaap6*, *Δaap8*, *Δaap9*, *Δaap10*, and *Δaap9Δaap10* generally showed phenotypic attributes not far from the *wt* with respect to oxidative and osmotic resistance, haploid fruiting, cell wall and membrane destabilizers, secretion of phospholipase, urease, melanin, and to some extent capsule formation; however, the stress implication of *Δaap4Δaap5* is always highly significant compare to the *wt* [9, 13]. Notably, *Δaap4Δaap5* is the only double mutated avirulent strain in an animal study reported [13]. This, therefore, makes Aap4 and Aap5 conspicuous and highly significant permeases in the survival and virulence of *C. neoformans*.

It is pertinent to quickly stress that *C. neoformans* var. *neoformans* is disadvantaged in metabolizing *D-*amino acids but *L-*stereotype due to evolutionary recessing *Dao* gene that encodes *D*-amino acid oxidases (Dao1, Dao2, and Dao3). *Dao* gene, however, is available in var. *gattii* and can metabolize *D-*amino acids as nitrogen sources. Interestingly, Aap4 is the only identified membrane transporter with *D-* and *L-*stereotype affinity with oxidase activity for Asp [19]. Notwithstanding, growth is usually and mostly denser in *L*-amino acids-containing media than in the corresponding *D*-amino acids media. Pathologically, *Δdao* mutants of *C. neoformans* are virulent, but *Δdao* mutants of *C. gattii* are attenuated. Again, it is uncertain if Gat1 controls *Dao* expression, unlike *Aap* in the absence/limited preferred nitrogen source.

## 7. Antifungal Drugs towards Cryptococcal Aaps

Targeting membrane transporters/permeases of infectious pathogens is one of the effective ways of impairing their growth and preventing further proliferation. This may become a challenge when such permease shares significant levels of homology with the host permeases. With *Cryptococcus*, the Aaps show functional redundancy, replaceability, synergism, and complementarity. The ten isoforms show appreciable levels of sequence homology, which are largely distant from humans. All cryptococcal Aaps show ≤40% sequence homology with humans, which indicates a predictable different supramolecular structure, an advantage that could be utilized in anti-cryptococcal drug design.

However, it is important to re-emphasize that such drug development will require a painstaking effort because a drug that interacts and binds a specific fungal Aap is needed rather than being promiscuously transported into the cells for intracellular action. Apart from the classical antifungal drugs that target fungi ergosterol biosynthesis and impose stress on the cell wall and membrane, developing therapeutical drugs against amino acids metabolism has been specifically centered around the critical regulatory steps in the metabolisms of essential amino acids exclusively unique to these fungi [62–65]. Thus, convincing evidence is needed to show selective and irreversible drug-Aaps affinity such that the supply channels are perpetually shut off. To buttress this, Garbe and Vylkova speculated that blocking committed steps in the metabolism of essential amino acids in pathogenic fungi could impel the fungi into commensal interaction with the infected host that may result in specialized expression of amino acid uptake mechanism, assuring a continual supply of nutrients within the infected host [66].

Futhermore, many years back, Pro transporter and branched-chain Aaps were shown to allow inducible transportation of icofungipen/cispentacin (a Pro analogue) into the cytoplasm of an invasive *C. albicans* to inhibit isoleucyl-*t*RNA synthase [67] and homoserine dehydrogenase [68] but this drug could only prevent the binding and inducible transportation of Pro competitively [67, 69], which means the drug and Pro compete for the permease. Good enough, Pro binding was shown to reduce by 75% in the presence of this drug [69]. Notwithstanding, Konishi et al. had earlier shown a weak activity of icofungipen against *C. neoformans* [70].

Targeting Aaps with ubiquitin-dependent permease endocytic drugs is a developing technique. One such is FTY720 (known as Fingolimod or 2-amino-2-[2-(4-octylphenyl)]-1,3-propanediol hydrochloride) [71, 72], first identified as a sphingosine analogue extracted from the fungus *Isaria sinclairii* and used to modulate sphingosine-1-phosphate receptor [73] but discovered to initiate promiscuous endocytosis of ubiquitin-dependent nutrient transporters [74] and most amino acid permeases [75]. This pharmaceutical advantage of FTY720 has been explored as an immunomodulator [71], promoting allograft survival [76] and significantly attenuating cancer cell proliferation by preventing essential amino acid uptake [77]. Another example is Sulfasalazine, a synthetic drug (salicylate + sulfa-drugs) that inhibits cysteine transporter [78, 79] and mostly used in bowel disease conditions but has not been explored against fungi Aaps. Extending the modulatory effects of these drugs on amino acid transporters of pathogenic *Cryptococcus* holds a promising antifungal therapy against this invasive pathogen; however, this needs to be done by moderating the parent drug for selective binding of pathogenic fungi Aaps.

## 8. Conclusions and Perspectives

*C. neoformans* remains one of the most adaptive opportunistic pathogenic fungi responsible for secondary morbidity infection in immunocompromised patients. Apart from other global transcriptional control factors, *C. neoformans* largely depend on the availability of sugars and NH_4_^+^ to determine the use of alternative carbon sources and amino acids. Ideally, carbons from sugars and low molecular weight organic acids are mainly preferred by *C. neoformans.* In this article, we reviewed a few numbers of available work on Aap relevant to *C. neoformans* survival and virulence and observed that *Aap1*, *Aap2*, *Aap4*, *Aap5*, *Aap8*, *Aap9*, and *Aap10* expressions are paramount to cryptococcal cell survival and virulence; however, the combined knocked-out of *Aap4* and *Aap5* is highly consequential.

Though only a few of these Aaps are critically articulated into the survival and virulence of cryptococcal cells, their regulations remain partially understandable. Apart from the CCR and NCR that determine the expression patterns of Aaps, GATA, GAAC, Gpp2, Hog1, and Ras1 are among the notable transcription factors that directly or indirectly modulate the expression and biological functions of these global permeases in cryptococcal cells. It is rather surprising, therefore, to observe how Aap function plummets in physiological temperature, notwithstanding thermotolerance and actinization promoted by the expression and activation of Ras1p through nutrient sensors, Cac1 and GTPases buffer heat-prone *C. neoformans* Aaps for survival, adaptation, and virulence, even in nonpermissive temperatures (Figure 1).

It is equally important to stress that drug targets against cryptococcal Aaps are prospective antifungal strategies that, if explored, may contribute to resolving the emerging antifungal resistance in cryptococcosis. Thus, studies that target virulence-induced membrane proteins necessary for fungi nutrient assimilation and survival should be embraced for new antifungal drug development; however, a careful consideration that will preclude the consequences in humans is highly recommended.

## Figures and Tables

**Table 1:** Expression of *C. neoformans* Aaps in different temperatures and supplemented media conditions.

Aap	1	2	3	4	5	6	7	8	9	10
30°C	ND	NE	ND	NE	NE	ND	ND	ND	NE	NE
37°C	Induced	NE	ND	Induced	Induced	NE	ND	NE	Induced	Repressed
39°C	ND	ND	ND	Induced	Induced	NE	ND	NE	ND	ND
Acidic amino acid	ND	ND	ND	Induced	NE	ND	ND	ND	ND	ND
Any N-source	ND	Induced	ND	Induced	Induced	NE	ND	NE	ND	ND
CCR/YPD (glucose)	Repressed	Repressed	NEx	Repressed	Repressed	Repressed	NEx	Repressed	Induced	Repressed
Eugenol	ND	ND	ND	Induced	Induced	ND	ND	ND	ND	ND
Fluconazole	ND	ND	ND	Induced	Induced	ND	ND	ND	ND	ND
Galactose	Induced	Induced	NEx	Induced^*∗*^	Induced^*∗*^	Induced	NEx	Induced	Induced	Induced
Lysine	NE	Induced	Induced^*∗*^	Repressed	Repressed	Induced	ND	Induced	Induced	Induced
NCR (NH_4_^+^)	Repressed	Repressed	Repressed^*∗*^	NE	Repressed	Repressed	NEx	Repressed	Repressed	Repressed
Nutritional stress	Induced	ND	NEx	Induced	Induced	Induced	NEx	Induced	ND	ND
*S-*amino acids	ND	ND	ND	ND	ND	NE	ND	ND	Induced	Induced
Try/Met/His	ND	Induced	NEx	Induced	Induced^*∗*^	NE	NEx	Induced	Induced	Induced
Tryptophan	Induced	Induced^*∗*^	NEx	ND	ND	NE	NEx	Induced	Induced	Induced

Based on the published data [9, 13, 19], we examined the probability of specific Aap gene expression or repression based on the survival of *C. neoformans* in a conditional medium when starved, exposed to antifungal, and cultured under varying temperatures. All amino acids are added to synthetic dextrose (SD). N-source = nitrogen source; CCR = carbon catabolite repression; ND = not detected; NE = no effect; NEx = no expression; YPD = yeast extract, peptone, dextrose (YEPD). ^*∗*^Most significantly and differentially expressed.

**Table 2:** *C. neoformans* growth conditions in SD media at 30°C with amino acids as sole nitrogen sources [9, 13].

Support >80% growth	Support <80% growth	Support <50% growth	Support <5% growth
Glycine	Serine	Valine	Alanine
Asparagine	Lysine	^*∗∗*^Leucine	Cysteine
Aspartate	^*∗∗∗*^Tryptophan	Isoleucine	Threonine
Glutamine	Phenylalanine	Methionine	Histidine
Glutamate	^*∗∗∗*^Proline		^*∗*^Tyrosine
	^*∗∗∗*^Arginine		

All growths are compared to NH_4_^+^-supplemented synthetic dextrose (SD). ^*∗*^Soluble at pH 2.0 but does not support cryptococcal cell growth. ^*∗∗*^Could support growth unpredictably more than NH_4_^+^. ^*∗∗∗*^Could be highly competitive sometimes with NH_4_^+^.

**Table 3:** Stress factors that influence GATA-transcriptional factors in *C. neoformans* [27–33].

Factors	Induced GATA	Repressed GATA	Targeted Aaps	Phenotypic traits
30 ⟶ 37°C	N/A	Gat1	Aap1, Aap2, Aap4, Aap5	Thermotolerance, virulence, mating
5-Fluocytocine	Bzp2	Gat204, Rim101	N/A	Antifungal resistance
Amphotericin	Bwc2, Bzp2, Gat201, Gat204, Rim101	N/A	N/A	Antifungal resistance
Cell wall and membrane stress agents	Bzp2, Gat1, Gat5, Gat6, Gat7, Gat201, Rim101	Bwc2	N/A	Survival, adaptation, and mating
ER-stress agents	Gat7, Gat201	Gat1, Gat6	N/A	Survival and adaptation
Fluconazole	Bzp2	Gat5, Gat7	Aap4, Aap5	Antifungal resistance
Fludioxonil	Bzp2	Gat7, Gat204	N/A	Antifungal resistance
Genotoxin (hydroxyurea)	Bzp2, Gat5, Gat6	N/A	Aap4, Aap5	Genotoxic resistance
Heavy metal	Bzp2, Gat5, and Tup1	Gat7, Gat201, Gat204	N/A	Survival and adaptation
Nitrogen uptake and pseudohyphal formation	Gat1	N/A	Amt1, Amt2	Virulence, invasive growth, and mating
Osmotic stress	Bzp2, Gat5, Gat6, Gat201, Gat204, Rim101	N/A	Aap4, Aap5	Survival, adaptation, and mating
Oxidative and nitrosative stress	Bzp2, Gat5, Gat6, Gat201, Gat204, Rim101	N/A	Aap4, Aap5	Survival, adaptation, and mating
Radiation	Gat5, Gat6, Bwc2, Bzp2	^*∗*^Gat1	Aap4, Aap5	Survival and virulence
Rapamycin	N/A	Gat1	Aap4, Aap5	Antifungal resistance
Rifampicin	Gat5, Gat6	^*∗*^Gat1	Aap4, Aap5	Antifungal resistance

^*∗*^Dispensable GATA transcription factor. N/A = not available.

**Table 4:** Significant phenotypic defects/advantages associated with *C. neoformans* mutants for nitrogen and amino acid uptake [9, 13, 21, 44, 45].

Permease mutants	Phenotypic defects/advantages
*Δaap1*	Growth reduction in Met and Pro supplemented SD media at 30/37°C
*Δaap1Δaap2*	Significant growth reduction in Gln and Arg at 30°C and in 60% of culturable amino acid media at 37°C
*Δaap2*	Wild-type growth in most amino acids supplemented SD but a significant growth reduction in Ile, Arg, and Lys at 30°C. Higher significant growth reduction at 37°C in most supplemented SD
*Δaap2*, *Δaap4*, *Δaap5*, *Δaap4Δaap5*	Mutants could not survive 5.0 mM H_2_O_2_ in YPD media but appeared to be restored in amino acid-supplemented SD except *Δaap4Δaap5* mutant. All displayed *wt* growth at 30°C regardless of the media, even at 37°C except *Δaap4Δaap5*
*Δaap2*, *Δaap4*, *Δaap5*, *Δaap9*, *Δaap10*, *Δaap4Δaap5*, *Δaap9Δaap10*	No survival in SD supplemented with Ala, Thr, Cys, His, or Tyr at 30°C
*Δaap2*, *Δaap4*, *Δaap5Δaap9Δaap10*	Normal growth at 30/37°C in any media
*Δaap4*	Virulent with *wt* growth in Leu, Asn, Glu, Gln, Arg, Trp, and Pro but a significant growth reduction in most other amino acid-supplemented SD at 30°C. Higher growth in most of the supplemented SD at 37°C. Hypersensitive to FCZ but not AmpB. Resistant to eugenol. Only mutant sensitive to *L-* and *D-*Asp-enriched SD media with impaired aspartate oxidase activity at translational and transcriptional levels
*Δaap4Δaap5*	Hypovirulent in *G. mellonella,* avirulent in MIMC, and survived with a significant growth reduction in most amino acid-supplemented SD but a *wt* growth in Gly and Met at 30°C. Further substantial growth reduction in ALL amino acid-supplemented SD at 37°C. Delayed capsule formation at 37°C. Hypersensitive to FCZ but not AmpB. Hyper-resistant to eugenol. Hypersensitive to as low as 1.0 mM H_2_O_2_
*Δaap5*	Virulent with *wt* growth in most amino acid-supplemented SD but a significant growth reduction in Gly, Ile, and Pro at 30°C. Further growth reduction at 37°C but less than *Δaap4*. Hypersensitive to FCZ but not AmpB. Resistant to eugenol
*Δaap6, Δaap8*	Wild-type growth at 30/37°C in ALL amino acid-supplemented liquid SD media for *Δaap6* but *Δaap8* showed a significant growth reduction in Met, Glu, and Trp at 37°C
*Δamt1*, *Δamt2*, *Δamt1Δamt2*	Formation of pseudohyphae in each single mutant but no pseudohyphae in the double mutant. Pseudohyphae displayed poor agar adherence and invasion—an attribute common to *Δras1* mutant. All mutants are virulent
*Δamt1/Δmep1*, *Δamt2*, *Δaap9Δaap10*	Homeostasis, nitrogen assimilation, alternative nitrogen source utilization, and virulence are impaired
*Δmep2/Δamt2*, *Δgpp1*, *Δgpp2*, *Δras1*	Sensitive to osmotic stress
*Δaap9*, *Δaap10*	Mutant survival is similar to the *wt* growth in most amino acid-supplemented SD but a significant growth reduction in Trp. *Δaap9* survived better in Pro at 30°C. Further substantial growth reduction in *Δaap10* than *Δaap9* at 37°C. ALL the mutants, including *Δaap9Δaap10*, showed *wt* growth under oxidative stress
*Δaap9Δaap10*	Displayed *wt* growth in most amino acid-supplemented SD but a significant growth reduction in Val, Ser, Met, Try, and Pro at 30°C. Further significant growth reduction in ALL the amino acid-supplemented SD at 37°C but *wt* growth in Leu, Ser, Glu, and Lys. The mutant also showed delayed capsule formation and production at 37°C

## Data Availability

All the literature data used to support the findings of this study are included within the article.
